# Pretreatment with millet-derived selenylated soluble dietary fiber ameliorates dextran sulfate sodium-induced colitis in mice by regulating inflammation and maintaining gut microbiota balance

**DOI:** 10.3389/fnut.2022.928601

**Published:** 2022-09-07

**Authors:** Weihao Wang, Fang Kou, Juan Wang, Zhigang Quan, Shuting Zhao, Yifei Wang, Xin Hu, Hunan Sun, Longkui Cao

**Affiliations:** ^1^College of Food Science, Heilongjiang Bayi Agricultural University, Daqing, China; ^2^National Coarse Cereals Engineering Research Center, Heilongjiang Bayi Agricultural University, Daqing, China; ^3^College of Life Science and Biotechnology, Heilongjiang Bayi Agricultural University, Daqing, China; ^4^Department of Marine Food Science and Technology, Gangneung-Wonju National University, Gangneung, South Korea

**Keywords:** millet, Se-SDF, colitis, inflammatory response, gut microbiome

## Abstract

Inflammatory activation and intestinal flora imbalance play key roles in the development and progression of inflammatory bowel disease (IBD). Soluble dietary fiber (SDF) and selenium have been proven to be effective for preventing and relieving IBD. This study investigated and compared the therapeutic efficacy of millet-derived selenylated-soluble dietary fiber (Se-SDF) against dextran sulfate sodium (DSS)-induced colitis in mice alone or through the synergistic interaction between selenium and SDF. In female mice, Se-SDF markedly alleviated body weight loss, decreased colon length, reduced histological damage scores, and enhanced IL-10 expression to maintain the barrier function of intestinal mucosa compared to male mice. The 16S rRNA sequence analysis further indicated that pretreatment with Se-SDF restored the gut microbiota composition in female mice by increasing the relative abundance of *Lactobacillus* and the Firmicutes/Bacteroidetes ratio. In conclusion, these findings demonstrated that Se-SDF can protect against DSS-induced colitis in female mice by regulating inflammation and maintaining gut microbiota balance. This study, therefore, provides new insights into the development of Se-SDF as a supplement for the prevention and treatment of colitis.

## Introduction

Colitis is a chronic disease that is associated with colorectal cancer (1–3). Patients with colitis usually present with diarrhea, abdominal pain, stools with mucus and pus, bloody stools, tenesmus, constipation, emaciation, and fatigue, which often recur as repeated attacks (4). In clinical practice, colitis can be treated using surgery or drugs, such as aminosalicylic acid preparations, glucocorticoids, and immunosuppressants (5, 6). However, the widespread application of these drugs has been limited by their side effects, which include mild-to-severe nausea, vomiting, edema, and increased blood glucose levels, as well as other complications, such as necrosis of the femoral head, osteoporosis, and impaired liver function (7, 8). It is, therefore, important to develop new therapeutic procedures based on natural biomolecules that can precisely regulate intestinal inflammation without destroying normal tissues.

Organic selenium (Se) is a dietary supplement that can exert significant therapeutic effects on intestinal inflammatory diseases (9) and is safer than inorganic Se. Unfortunately, organic Se is less abundant in nature (10), and it is time-consuming to produce Se-rich natural products through biotransformation (11). Chemical modification is a rapid, safe, and effective strategy for producing natural Se-rich products to prevent or treat intestinal inflammatory diseases and regulate the intestinal barrier and gut microbiota. For instance, numerous studies utilized chemical modification to generate Se-enriched natural products and reported that the derivatives of natural biomolecules can display significant biological activities after appropriate molecular modification (11). Indeed, Zhu et al. combined Se and *Ulva lactuca* polysaccharide and showed that not only it reduces the toxicity of inorganic Se, but it also protects against DSS-induced acute colitis in mice by mitigating the loss in body weight and inflammatory damage in the colon (9, 12). The preparation of effective, natural Se-rich products through chemical modification is, therefore, an interesting prospect for therapeutic applications.

Soluble dietary fiber (SDF) is a unique polysaccharide that can help to minimize or prevent chronic inflammation while modulating the mucosal immune system, improving intestinal barrier function, and maintaining the balance of beneficial bacteria in the human intestine (13–15). Research studies have suggested that SDF can decrease the production of inflammatory cytokines and intestinal membrane permeability in order to reduce inflammation, which is associated with the breakdown and fermentation of dietary fiber by gut microbiota. This subsequently increases the production of short-chain fatty acids, such as acetate, propionate, and butyrate, and promotes the release of inflammatory cytokines by Bacteroidetes and Firmicutes, leading to changes in bacterial abundance (16). Millet SDF is a by-product of millet processing that has been reported to enhance fecal bulk, stimulate the gastrointestinal tract, and reduce blood glucose levels, cholesterol levels, and intestinal inflammation (17). However, millet SDF has a low comprehensive utilization rate, and it is mainly used for animal feed or discarded as waste, which can cause environmental pollution. The development of a natural bioactive prebiotic using millet SDF would, therefore, not only improve the value and comprehensive utilization rate of millet, but also avoid wasting resources.

Previous studies have reported the modification of millet SDF; however, the ability of selenylated-soluble dietary fiber (Se-SDF) to treat and prevent colitis by regulating the structure of the human intestinal microbiome remains unclear. In this study, we employed a mouse model of dextran sulfate sodium (DSS)-induced colitis to verify the possible relationship between Se-SDF and the amelioration or prevention of colitis. This study, therefore, improves our understanding of chemically modified SDF and provides a new therapeutic strategy for using dietary Se supplementation to prevent and treat colitis.

## Materials and methods

### Materials

Millet was obtained from the National Coarse Cereals Engineering Research Center (Heilongjiang Bayi Agricultural University, Daqing, China). Basic feed was purchased from TROPHIC Animal Feed High-Tech Co., Ltd. (feed code: TP23300; Beijing, China). DSS was purchased from MP Biomedicals (California, United States). All other chemicals and reagents were of analytical grade (> 99.9% purity).

### Selenylated-soluble dietary fiber preparation

Millet SDF was prepared using an enzymatic method, as described previously (18). Briefly, defatted millet powder was mixed (1:50, w/v) with 0.08 mol/L phosphate-buffered saline (PBS, pH = 6), and then high-temperature resistant α-amylase, protease, and amyloglucosidase were added to extract millet water SDF at 100°C for 10 min. The supernatant was concentrated using a vacuum rotary evaporator, centrifuged at 5,000 × *g* for 10 min, and precipitated using 95% ethanol (1:4, v/v). The precipitate was dried at –108°C using a vacuum freeze dryer and stored at 4°C before use.

Se-SDF was prepared using nitric acid solution using the sodium selenite method with minor modifications (19, 20). Briefly, SDF was mixed with 0.5% nitric acid solution (1:100, w/v), barium chloride solid powder (1:1.3, w/w), and 5 mg/mL sodium selenite solution (1:6, w/v) before being incubated at 40°C for 6 h. After the mixture had been cooled to 20°C, the pH was adjusted to 5–6 units using saturated sodium carbonate solution and sodium sulfate was added to remove Ba^2+^. The mixture was centrifuged at 5,000 × *g* for 10 min and the supernatant was concentrated, dialyzed with distilled water using a dialysis tube (3,500 MWCO), and dried using a vacuum freeze dryer at –108°C. Se-SDF was stored at 4°C before use. The content of selenium in Se-SDF was 2.69 mg/g, the molecular weight was 1.09 × 10^4^ Da, and the characteristic absorption peaks of Se-SDF including Se-O-C (600 cm^–1^) and Se = O (832 cm^–1^), Se-OH (695 cm^–1^), respectively.

### Experimental animals and design

Seventy specific-pathogen-free (SPF) BALB/c mice (35 female and 35 male, 7-week-old, 18–20 g) were obtained from the Changchun Yisi Laboratory Animal Technology Co., Ltd. (Changchun, China). The mice were housed in an SPF environment at an ambient temperature of 26 ± 1 °C with 50–70% humidity and were given standard chow and distilled water *ad libitum*, with 1 week of acclimation before the experiments. Animal feeding and treatment protocols were conducted according to the guidelines of the Chinese Association for Laboratory Animal Sciences (CALAS) and all animals were maintained in strict accordance with the Chinese regarding animal experimentation.

**FIGURE 1:**
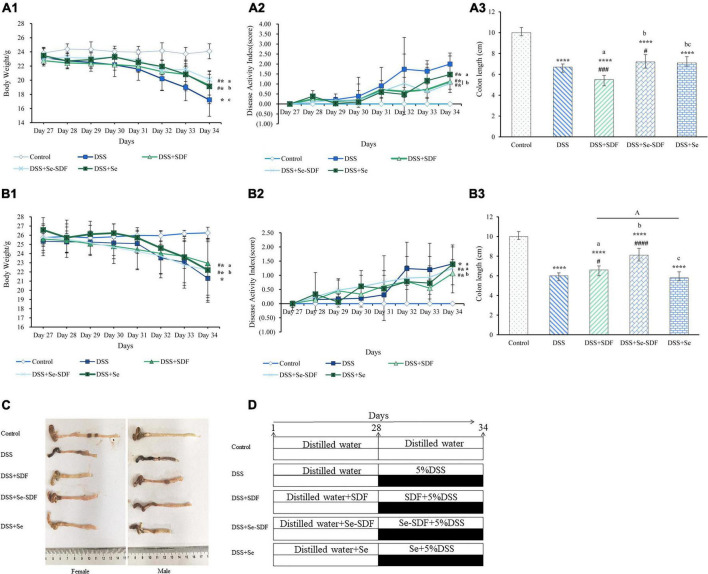
Effect of Se-SDF on clinical symptoms in mice with DSS-induced colitis. **(A_1_–A_3_)** Body weight (g), disease activity index (DAI), and colon length (cm) of female mice. **(B_1_–B_3_)** Body weight (g), DAI, and colon length (cm) of male mice. **(C)** Representative images of the colon from each group. **(D)** Schematic representation of the experimental design. Data are expressed as mean ± SD (*n* = 3). *p < 0.05, **p < 0.01, ***p < 0.001, ****p < 0.0001 vs. control group. ^#^*p* < 0.05, ^##^*p* < 0.01, ^###^*p* < 0.001, ^####^*p* < 0.0001 vs. DSS group. Different letters indicate significant differences between groups (*p* < 0.05).

The mice were randomly assigned to five groups (Figure 1D; *n* = 14 per group with 7 females and 7 males): control, DSS, DSS + SDF, DSS + Se-SDF, and DSS + Se. All groups were fed a normal diet and distilled water for 35 days from 08:00 p.m. to 08:00 a.m. The SDF (14.09 mg/kg) and Se (Se 38 μg/kg) groups were orally administered with 0.31 mg/mL of SDF solution and 0.002 mg/mL of sodium selenite solution for 34 days, respectively. The Se-SDF (SDF 14.09 mg/kg, Se 38 μg/kg) group was fed 0.31 mg/mL of Se-SDF solution for 34 days. All groups except the control group were treated with 5% DSS (M.W.: 40,000 Da) on day 28 to establish the colitis model.

All mice were sacrificed by cervical dislocation at the end of the experimental period (day 35) and their colons were collected for histological analysis. Colon contents were subjected to biochemical analysis and serum cytokine levels were quantified. Blood samples were centrifuged (14,000 × *g* for 10 min at 4°C) and serum was stored at –80°C until use.

### Assessment of disease activity index

To assess the severity of DSS-induced colitis, we monitored body weight, hemoccult test results or rectal bleeding, and stool consistency in the last 7 days (21). The disease activity index (DAI) was calculated as described in Supplementary Table 1.

### Histological injury and hematoxylin and eosin staining

A sample from the mid-section of the colon was fixed in 4% paraformaldehyde, embedded in paraffin, sectioned (4 μm), and stained with hematoxylin and eosin (H&E) (9). The degree of colitis was assessed as described in Supplementary Table 2. Colon contents were cooled with liquid nitrogen and stored at –80°C prior to analysis.

### Enzyme-linked immunosorbent assay

Serum cytokines, including IL-6, TNF-α, and IL-10, were detected using an enzyme-linked immunosorbent assay (ELISA) kit according to the manufacturer’s protocol (Shanghai Jianglai Biological Technology, Shanghai, China).

### 16S rRNA sequencing analysis

Total bacterial DNA was extracted from the colon using a DNA extraction kit (QIAamp DNA Stool Mini Kit, Qiagen, Inc., Shanghai, China) as described previously (22) using the following 16S rDNA V3 to V4 region primers: V3, 357F 5′-ACTCCTACGGRAGGCAGCAG-3′; V4, 806R 5′-GGACTACHVGGGTWTCTAAT-3′. An Ion A Torrent PGM system was used for on-machine sequencing and the results were filtered using FASTX Toolkit 0.0.13 software. The RDP Classifier Bayesian algorithm was used to classify 97% of the similar operational taxonomic unit (OTU) representative sequences and to determine the community composition of each sample at the genus and species levels. The similarity between samples was calculated according to the difference in OTU composition and abundance in each sample. Principal coordinate analysis (PCoA) was performed using the TAYc algorithm to detect individual differences between intestinal bacteria in each group (23).

### Statistical analysis

All data were expressed as the mean ± SD of triplicate experiments and were analyzed using GraphPad Prism 8.0 software (GraphPad Software Inc., San Diego, CA, United States). and SPSS 19.0 software (SPSS, Chicago, IL, United States). Differences were considered significant at *p* < 0.05.

## Results

### Effect of selenylated-soluble dietary fiber on dextran sulfate sodium-induced colitis in mice

First, we measured changes in the body weight of mice in each group (Figure 1) in the last 7 days of the experiment after DSS administration to determine their physical condition. In the female mice, body weight was significantly decreased in all treatment groups compared to the control group (*p* < 0.0001); however, Se-SDF treatment was able to restore body weight loss to a greater degree than the other treatments (Figure 1A1). Similar results were observed in the male mice (Figure 1B1), except that the SDF treatment had better protective effects than Se-SDF.

The DAI was assessed in both the female and male mice based on their body weight loss, stool consistency, and occult/gross bleeding after 27 days (Figures 1A2,B2). Female mice in the DSS + Se-SDF and DSS + SDF groups had a lower DAI than those in the DSS group, albeit with no significant difference. Male mice in all the groups with DSS-induced colitis also exhibited markedly higher DAI scores than those in the control group (Figure 1B2), with the DSS + SDF group displaying a lower DAI than the DSS + Se-SDF group. Thus, both SDF and Se-SDF were able to reduce intestinal damage in female mice, whereas SDF reduced the symptoms of colitis more than Se-SDF and Se in male mice.

The colons of female mice were significantly shorter in all DSS-induced colitis models than in the control group (*p* < 0.001); however, colons were longer in the DSS + Se-SDF and DSS + Se groups than in the DSS + SDF group (Figures 1A3,C), suggesting that Se-SDF and Se may protect against colon shortening in female mice. Similarly, male mice in the DSS + Se-SDF group displayed remarkably less colon shortening than those in the DSS-induced colitis model (Figures 1B3,C). Therefore, Se-SDF appears to effectively prevent colon shortening in both female and male mice with DSS-induced colitis.

Taken together, the observed changes in the body weight, colon length, and DAI of female mice suggest that Se-SDF can effectively relieve the symptoms of DSS-induced colitis, whereas SDF was able to more effectively mitigate weight loss and reduce the DAI in male mice.

### Effect of selenylated-soluble dietary fiber on histological changes in dextran sulfate sodium-induced colitis

**FIGURE 2:**
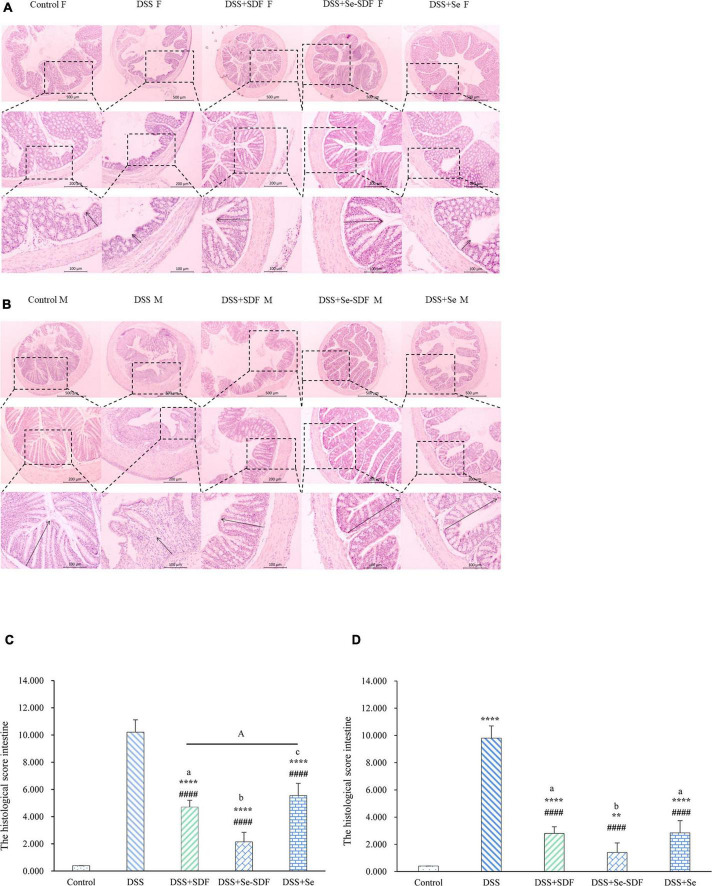
Effect of Se-SDF on histopathological changes in mice with DSS-induced colitis. **(A)** Histological scores of female mice. **(B)** Histological scores of male mice. **(C)** Histological analysis of female mice. **(D)** Histological analysis of male mice. Data are expressed as mean ± SD (*n* = 3). *p < 0.05, **p < 0.01, ***p < 0.001, ****p < 0.0001 vs. control group. ^#^*p* < 0.05, ^##^*p* < 0.01, ^###^*p* < 0.001, ^####^*p* < 0.0001 vs. DSS group. Different letters indicate significant differences between groups (*p* < 0.05).

Next, we performed histological analysis on colon samples from mice in each treatment group (Figures 2A,B). Microscopic inflammation and extensive colonic tissue damage, including inflammatory cell infiltration, crypt damage, and focus formation, were observed in mice with DSS-induced colitis. In addition, colons from mice treated with DSS displayed shortened intestinal villi, incomplete villus structures, and the proliferation of lymphoid follicles with disrupted intestinal endothelial structures. The colons of both female and male mice from the DSS + Se-SDF, DSS + Se, and DSS + SDF groups had restored colon tissue damage with longer ileac villi and more complete villus structures than that in the DSS group. In addition, male and female mice from the DSS + Se-SDF, DSS + Se, and DSS + SDF groups had significantly lower intestinal histological scores than those from the DSS groups (Figures 2C,D). The histological scores of female: DSS + Se-SDF (2.1), DSS + Se (5.3), and DSS + SDF(4.4); male: DSS + Se-SDF (1.4), DSS + Se (2.2), and DSS + SDF(2.5); *p* < 0.0001. Together, these findings indicate that Se-SDF, SDF, and Se can inhibit colon tissue damage and inflammatory responses in the following order: Se-SDF > SDF > Se.

### Effect of selenylated-soluble dietary fiber on inflammatory cytokine expression

**FIGURE 3:**
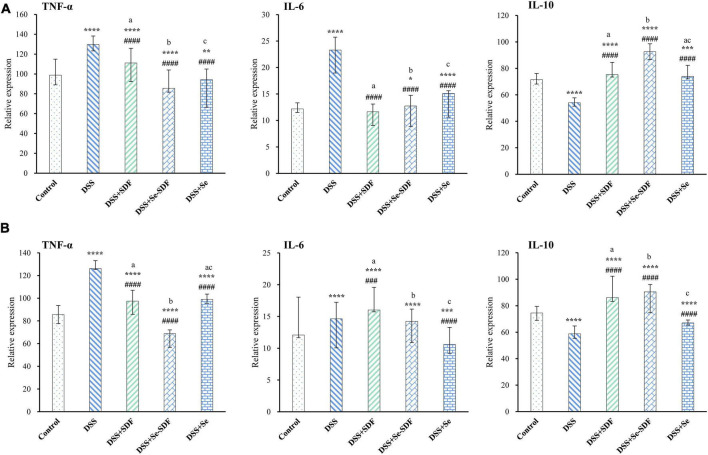
Effect of Se-SDF on inflammatory factors in mice with DSS-induced colitis. **(A)** Inflammatory factors in female mice. **(B)** Inflammatory factors in male mice. Data are expressed as mean ± SD (*n* = 3). *p < 0.05, **p < 0.01, ***p < 0.001, ****p < 0.0001 vs. control group. ^#^*p* < 0.05, ^##^*p* < 0.01, ^###^*p* < 0.001, ^####^*p* < 0.0001 vs. DSS group. Different letters indicate significant differences between groups (*p* < 0.05).

Inflammation is an important clinical manifestation of colon injury. Compared to that in the control group, DSS increased the expression of pro-inflammatory cytokines (TNF-α and IL-6) and decreased the expression of anti-inflammatory cytokines (IL-10) in female mice. Conversely, TNF-α and IL-6 expression were decreased in the DSS + Se-SDF, DSS + SDF, and DSS + Se groups and IL-10 expression significantly increased (*p* < 0.0001) compared to that in the DSS group (Figure 3A), with the DSS + Se-SDF group displaying higher IL-10 levels and lower TNF-α levels than that by the DSS + SDF and DSS + Se groups. Similar results were obtained for the male mice (Figure 3B), in which TNF-α levels were significantly decreased (*p* < 0.0001) and IL-10 levels were increased in the DSS + Se-SDF group compared to that in the DSS + SDF and DSS + Se groups. Taken together, these results suggest that Se-SDF could inhibit the pathogenesis of colitis in mice by promoting the release of anti-inflammatory cytokines (IL-10 in male and female mice) and suppressing pro-inflammatory cytokines (TNF-α and IL-6 in female mice, TNF-α in male mice).

### Effect of selenylated-soluble dietary fiber on gut microbiota diversity

**FIGURE 4:**
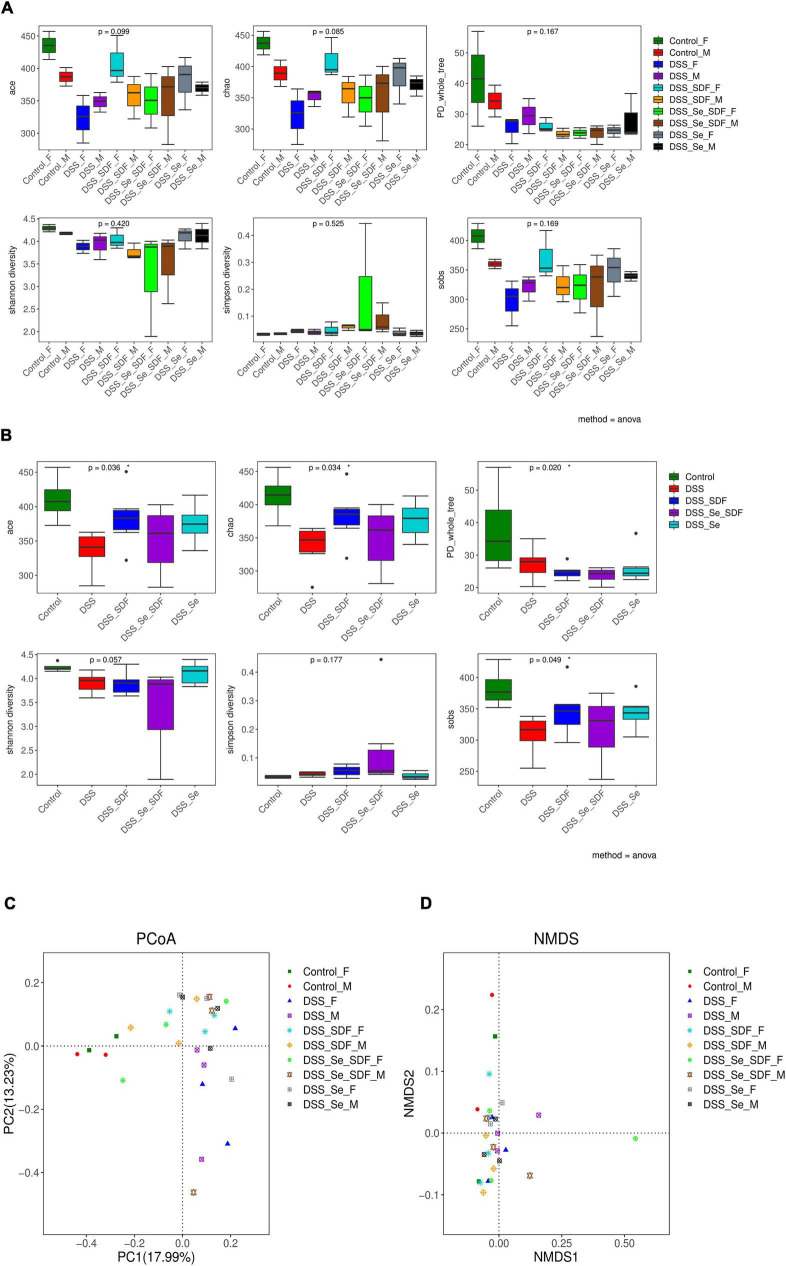
Effects of Se-SDF on gut microflora diversity in mice with DSS-induced colitis. Analysis of α- diversity in male and female mice **(A)** and the overall analysis **(B)**. Analysis of β- diversity of PCoA in male and female mice **(C)** and the NMDS analysis **(D)**.

Next, we examined the α-diversity of intestinal microbiota in colon samples from each group of mice using 16S rRNA gene sequencing. The intestinal microbiota compositions of both the female and male mice are shown in Figure 4A, and the overall analysis of each group is shown in Figure 4B. The community richness and diversity of colonic microbiota over the V3–V4 region were determined using the Ace, Chao, PD_whole_tree, Shannon, Simpson, and Sobs indices. Although no statistical differences were observed between the six indices in the female and male mice (Figure 4A), the community richness (Ace and Chao indices) and community diversity (Shannon and Simpson indices) were higher in the DSS + Se-SDF group than in the other groups. Compared to the control group, the overall analysis revealed that the Ace, Chao, PD_whole_tree, and Sobs indices were significantly different (*p* < 0.05) for the model groups (Figure 4B). Thus, Se-SDF, Se, and SDF appear to increase the α-diversity of gut microbiota in the following order: Se-SDF > SDF > Se.

The trends of beta diversity for DSS-induced male and female model groups were analyzed by PCoA to determine the overall differences in the gut microbial communities. As shown in Figure 4C, the microbial community in PC1 and PC2 were 17.99 and 13.23%, respectively, with no significant differences between the groups. The control groups for both females and males are farther apart on PC 1; however, the SDF, Se, and Se-SDF groups for both females and males are farther apart on PC 2, which indicates that the SDF, Se, and Se-SDF groups have a remarkable difference in PCoA 2. The non-metric multidimensional scaling (NMDS) analysis (Figure 4D) showed that the female SDF and Se-SDF groups presented significant differences in the abundance of gut microbial communities, while the others were similar. This result indicates that Se-SDF treatment effectively alters intestinal microflora diversity in female mice compared to that in male mice.

### Effect of selenylated-soluble dietary fiber on gut microbiota composition

**FIGURE 5:**
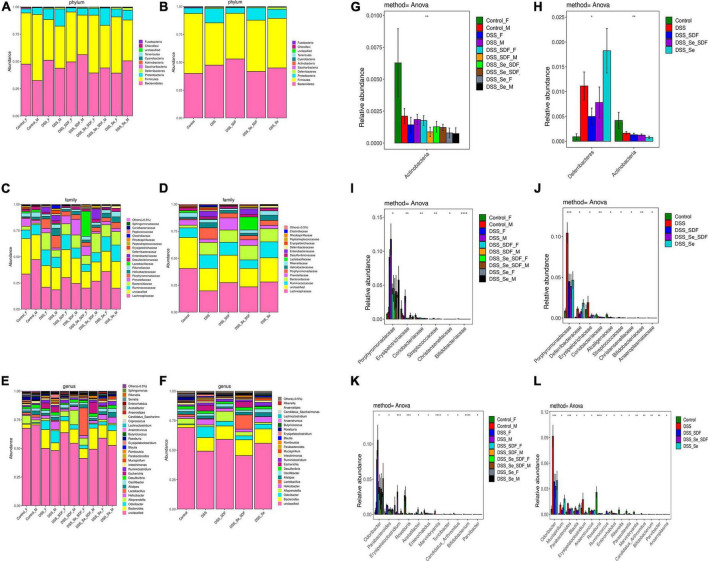
Microbial taxonomic composition and bacterial taxonomic comparison. Analysis of microbial taxonomic composition and community differences at the phylum level in female and male groups **(A,G)** and the overall analysis of **(B,H)**. Analysis of microbial taxonomic composition at the family level in female and male groups **(C,I)** and the overall analysis of **(D,J)**. Analysis of microbial taxonomic composition at the genus level in female and male groups **(E,K)** and the overall analysis **(F,L)**. *p < 0.05, **p < 0.01, ***p < 0.001, ****p < 0.0001.

Finally, we examined the microbial taxonomic composition and compared bacterial taxonomy at the phylum level in each of the different groups. As shown in Figure 5A, Firmicutes, Bacteroidetes, and Proteobacteria were the predominant bacterial communities in healthy male and female mice; however, the DSS groups had a decreased abundance of Firmicutes and Actinobacteria and increased abundance of Bacteroidetes and Proteobacteria compared to that in the control group (Figures 5A,G). Se-SDF and Se treatments markedly increased the Firmicutes/Bacteroidetes ratio in female mice. SDF treatment also restored the imbalanced colony structure induced by DSS and produced a similar bacterial composition to that observed in healthy mice at the phylum level. In male mice, SDF and Se treatments increased the abundance of Bacteroidetes and decreased the abundance of Proteobacteria. The overall analysis of each group at the phylum level revealed that DSS treatment markedly decreased Firmicutes richness while increasing the abundance of Bacteroidetes and potentially pathogenic Proteobacteria and Deferribacteres, suggesting that DSS induces colitis by enhancing pathogenic bacteria (Figures 5B,H). Se-SDF and Se administration reversed the changes observed in the DSS-induced colitis model by increasing the Firmicutes/Bacteroidetes ratio and decreasing Proteobacteria and Actinobacteria richness; however, SDF increased the abundance of Bacteroidetes and Firmicutes but decreased that of Proteobacteria (Figures 5B,H). Together, these findings indicate that both Se-SDF and Se can improve DSS-induced colitis by increasing the Firmicutes/Bacteroidetes ratio and decreasing Proteobacteria to balance intestinal microbiota, whereas SDF increases the abundance of Firmicutes and Bacteroidetes and decreases Proteobacteria to restore intestinal barrier function.

At the family level, DSS treatment decreased the abundance of Lachnospiraceae and Prevotellaceae in male and female mice but increased the abundance of Lactobacillaceae, Erysipelotrichaceae, and Porphyromonadaceae (Figures 5C,I). Although Se-SDF increased the abundance of Lachnospiraceae and Prevotellaceae and decreased that of Porphyromonadaceae in both male and female mice, the abundance of Lactobacillaceae increased only in females and that of Bacteroidaceae and Enterobacteriaceae increased only in males. The overall analysis of each group at the family level revealed that DSS treatment decreased the abundance of Lachnospiraceae and Prevotellaceae compared to that in the control group while increasing that of Porphyromonadaceae, Lactobacillaceae, and Enterobacteriaceae (Figures 5D,J and Table 1). Se-SDF treatment increased the abundance of Lactobacillaceae, Bacteroidaceae, and Lachnospiraceae but decreased that of Ruminococcaceae and Porphyromonadaceae, whereas SDF increased the abundance of Lachnospiraceae and Prevotellaceae and Se increased the abundance of Lachnospiraceae and Ruminococcaceae.

**TABLE 1:** Abundance of intestinal bacteria.

Intestinal bacteria	Control	DSS	DSS + SDF	DSS + Se-SDF	DSS + Se
Lachnospiraceae	0.40 ± 0.010	0.20 ± 0.009 *	0.27 ± 0.004	0.23 ± 0.002	0.28 ± 0.003
Ruminococcaceae	0.09 ± 0.001	0.12 ± 0.000*	0.12 ± 0.001*	0.08 ± 0.000	0.15 ± 0.004*
Bacteroidaceae	0.02 ± 0.000	0.12 ± 0.004*	0.11 ± 0.000*	0.13 ± 0.002*	0.12 ± 0.001*
Prevotellaceae	0.05 ± 0.002	0.03 ± 0.000	0.11 ± 0.007*	0.05 ± 0.001	0.03 ± 0.000
Lactobacillaceae	0.02 ± 0.000	0.01 ± 0.000	0.00 ± 0.000	0.12 ± 0.004*	0.00 ± 0.000
Desulfovibrionaceae	0.03 ± 0.001	0.02 ± 0.001	0.02 ± 0.000	0.03 ± 0.001	0.02 ± 0.000
Enterobacteriaceae	0.00 ± 0.001	0.05 ± 0.000	0.00 ± 0.000	0.05 ± 0.002	0.01 ± 0.000
Peptostreptococcaceae	0.01 ± 0.000	0.01 ± 0.000	0.00 ± 0.000	0.01 ± 0.000	0.00 ± 0.000
Rhodospirillaceae	0.00 ± 0.000	0.00 ± 0.000	0.00 ± 0.000	0.01 ± 0.000	0.00 ± 0.000
Clostridiaceae	0.00 ± 0.000	0.01 ± 0.000	0.00 ± 0.000	0.00 ± 0.000	0.00 ± 0.000

Data are expressed as mean ± SD (n = 3).

*p < 0.05 vs. control group.

Consistent results were also shown at the level of genus. In Figures 5F,L, the DSS treatment increased the abundance of pathogenic bacteria, including *Bacteroides*, *Odoribacter*, *Parabacteroides*, *Helicobacter*, *Erysipelatoclostridium*, and *Escherichia*, in male and female mice compared to that in the control group. Se-SDF treatment increased the abundance of *Lactobacillus* and decreased *Bacteroides* in female mice, while the abundance of *Bacteroides* and *Escherichia* increased in male mice (Figures 5E,K). The overall analysis of each group at the genus level revealed that only the Se-SDF group increased the abundance of *Lactobacillus* and Se treatment increased the abundance of *Ruminiclostridium* (Figures 5F,L), decreased *Parabacteroides*, and potentially pathogenic effectively. In conclusion, Se-SDF treatment alters specific bacteria in gut microbiota.

Taken together, these findings indicate that DSS induces colitis in mice by increasing the relative abundance of pro-inflammatory Proteobacteria taxa, including Enterobacteriaceae, while decreasing the abundance of Lachnospiraceae and Prevotellaceae. Se-SDF was able to reduce the proportion of pro-inflammatory bacteria and upregulate Lactobacillaceae and Bacteroidaceae to maintain a balanced gut microbiota. Similarly, SDF protected intestinal barrier function and intestinal microbiota composition by increasing the abundance of Prevotellaceae, which are beneficial bacteria, whereas Se increased the abundance of Lachnospiraceae and Ruminococcaceae.

## Discussion

Organic Se is an important trace element that has various physiological functions, including key roles in protein synthesis, and exerts significant therapeutic effects against colitis. In this study, we demonstrated that Se-SDF maintains and/or promotes intestinal health by decreasing inflammation and modulating the composition of intestinal microbiota in mice with DSS-induced colitis by reducing the expression of pro-inflammatory cytokines and promoting anti-inflammatory cytokines, which subsequently increases the abundance of beneficial bacteria and suppresses harmful bacteria.

After 3 days of DSS administration, the mice showed signs of diarrhea, anal bleeding, and weight loss, indicating that the model of DSS-induced colitis had been generated successfully (24). The administration of Se-SDF, SDF, and Se alleviated clinical symptoms and histological abnormalities in the mice with DSS-induced colitis, including preventing body weight loss and colon shortening. Interestingly, Se-SDF had the best protective effect followed by SDF and Se, suggesting that these prebiotic supplements could be used to prevent or mitigate the development of colitis. It is important to note, however, that the therapeutic effect of Se-SDF treatment differed slightly between male and female mice. For example, female mice in the DSS + Se-SDF group displayed significantly less weight loss, colon shortening, and a lower DAI, whereas in male mice these effects were more prominent in the DSS + SDF group. This difference may be due to metabolic differences caused by different hormone levels between female and male mice.

Inflammatory cytokines play crucial roles in the development and pathogenesis of colitis. In particular, the expression of the pro-inflammatory cytokines TNF-α and IL-6 correlates positively with the severity of colitis (25–28). The anti-inflammatory cytokine IL-10 is secreted by various immune cells, including macrophages, dendritic cells, and T cells, which play a protective role by inhibiting the Th1/Th17 pathogenic response and is therefore essential for maintaining intestinal barrier function (29). In this study, we found that Se-SDF strongly modulated diverse inflammatory mediators in both male and female mice with DSS-induced colitis. Se-SDF reduced the overproduction of pro-inflammatory cytokines by downregulating TNF-α and IL-6 expression and upregulating the expression level of IL-10, with SDF and Se exerting similar effects but to a lesser degree. These findings are consistent with the observed clinical symptoms and histological abnormalities, wherein Se-SDF synergistically reduced the local inflammatory response in mice with colitis and inhibited inflammatory cell infiltration and pathological damage in the colon (30). Significant differences in inflammatory cytokine expression were also observed between male and female mice: Se-SDF, SDF, and Se significantly increased the expression of the anti-inflammatory cytokine IL-10 and decreased IL-6 levels in female mice compared to that in male mice. Thus, these differences in cytokine regulation may underlie the anti-inflammatory efficacy of Se-SDF in female mice.

Gut microflora is an important component of the intestinal tract that influences intestinal health and can lead to intestinal inflammation if their dynamic balance is disrupted (31, 32). Prebiotics, such as Se and SDF, could improve the clinical outcome of colitis by improving the composition and abundance of gut microbiota to repair intestinal microbial dysbiosis. Herein, we investigated the α- and β-diversity of gut microbiota in mice with DSS-induced colitis. Both the community richness (Ace and Chao indices) and community diversity (Shannon and Simpson indices) were increased in male and female mice treated with Se-SDF, suggesting that Se-SDF can improve the abundance and diversity of intestinal microbiota. However, the β-diversity result indicates that Se-SDF treatment alters the intestinal microflora more effectively in female mice when compared to that in male mice.

Intergroup analysis of gut microbiota revealed that Se-SDF altered the DSS-induced imbalance in female mice by increasing the abundance of *Lactobacillus* and the Firmicutes/Bacteroidetes ratio while upregulating the expression of anti-inflammatory cytokines to reduce intestinal damage. In male mice, Se-SDF increased the abundance of Bacteroidetes to decrease the expression of the pro-inflammatory cytokine TNF-α. The overall analysis further revealed that Se-SDF, SDF, and Se increased the abundance of beneficial bacteria, such as Firmicutes and Lachnospiraceae; however, the following differences were observed: Se-SDF increased the abundance of Lactobacillaceae and Bacteroidaceae but decreased that of Ruminococcaceae; Se decreased the abundance of Ruminococcaceae; and SDF increased the abundance of Prevotellaceae and decreased that of Porphyromonas. Thus, Se, SDF, and Se-SDF could be used as prebiotics to effectively improve the composition and diversity of beneficial gut microbiota communities. Consistently, studies have proposed that DSS-induced colitis could be alleviated by enhancing the abundance of Lachnospiraceae, *Prevotella*, and Bacteroides, which have beneficial metabolic profiles and facilitate the recovery of the intestinal mucosal barrier by breaking down SDF to produce high levels and different proportions of short-chain fatty acids, including butyric acid (33, 34). Furthermore, a high abundance of *Lactobacillus* could reduce the permeability of intestinal mucosa in mice with colitis and decrease inflammatory cytokine expression to alleviate inflammation (35–37). It has also been reported that Se can exert anti-inflammatory and potentially beneficial effects to ameliorate experimental colitis (38) by modulating gut microbiota composition. Indeed, Li et al. found that selenylated α-D-1,6-glucan could prevent and treat DSS-induced colitis by decreasing the expression of inflammatory cytokines (TNF-α, IFN-γ, and IL-1β), upregulating IL-10 in the NF-κB and NLRP3 signaling pathways, and enhancing the abundance of beneficial bacteria (Bacteroidetes, Firmicutes) to improve the composition of intestinal microbiota (39). Similar results have also been reported by Deng et al. who proposed that Se@Albumin nanoparticles can decrease the expression of inflammatory cytokines and increase the abundance of Bacteroidaceae to ameliorate intestinal mucositis (40). Together with our findings, these data suggest that Se-SDF can protect intestinal function by inducing anti-inflammatory effects and regulating the structure and composition of intestinal microflora.

Nevertheless, this study has several limitations that should be considered in future research. Firstly, the efficacy of Se-SDF against intestinal inflammation was only investigated in mice; therefore, future studies should explore its effects in humans. Secondly, we found that Se-SDF altered the composition of the intestinal microflora; however, we did not analyze its effect on their metabolites. Lastly, although our study investigated the ability of Se-SDF to alleviate DSS-induced colitis by modulating the composition of intestinal flora and exerting anti-inflammatory effects, the underlying metabolic pathways and mechanisms remain unclear. Therefore, the subsequent research will explore these questions in-depth.

## Conclusion

In summary, this study demonstrated that Se-SDF dramatically reversed the loss in body weight and colon shortening induced by DSS in female mice while suppressing the expression of pro-inflammatory cytokines (IL-6 and TNF-α) and upregulating anti-inflammatory cytokines (IL-10). Importantly, Se-SDF was able to maintain intestinal barrier function and regulate the composition of gut microbiota by increasing the abundance of *Lactobacillus* and the Firmicutes/Bacteroidetes ratio and inhibiting harmful bacteria. Thus, Se-SDF could be a useful prebiotic for regulating the intestinal barrier in colitis, particularly in females. This study fills a research gap regarding Se-modified dietary fiber, while providing a new therapeutic strategy for the use of dietary Se supplementation to prevent and treat colitis.

## Data availability statement

The sequencing data has been uploaded to the public repository. The Bio project number is PRJNA833531 and the sequence number is SRP372932.

## Ethics statement

The animal study was reviewed and approved by the Chinese Association for Laboratory Animal Sciences (CALAS).

## Author contributions

WW provided experimental plans. JW performed the experiments and was responsible for data acquisition. FK was responsible for writing the original draft, methodology, and review. ZQ, SZ, YW, and XH helped to analyze data and contributed toward writing the manuscript. HS and LC were responsible for conceptualization, methodology, supervision, and funding acquisition. All authors have read and agreed to submit the manuscript.
