# Supplementary material for: Pretreatment with millet-derived selenylated soluble dietary fiber ameliorates dextran sulfate sodium-induced colitis in mice by regulating inflammation and maintaining gut microbiota balance
Source: Front Nutr. 2022 Sep 7;9:928601. doi: 10.3389/fnut.2022.928601 (PMC9494682; doi:10.3389/fnut.2022.928601)
Supplement: Supplementary file 1 [file Data_Sheet_1.pdf]

Pre-Administration of Selenylation Soluble Dietary Fiber from Millet Ameliorates DSS-Induced Colitis in Mice via Regulating Inflammation and Maintaining Gut Microbiota Balance

Table S1 Disease activity index (DAI) evaluation criteria.

Score	Weight Loss (%)	Stool Consistency	Occult/Gross Bleeding
0	None	Normal	Negative
1	1-5		
2	5-10	Loose stool	Positive
3	10-20		
4	>20	Diarrhea	Gross bleeding

Table S2 Histology injury score chart.

Grade	0	1	2	3	4
Inflammation	None	Mild	Moderate	Severe	-
Mucosal damage	None	Mucous layer	Submucosa	Muscularis and serosa	-
Crypt damage	None	1/3	2/3	100%	100% with epithelium loss
Pathological change range	None	0%–25%	26%–50%	51%–75%	76%–100%
